# Quantitative mapping and spectroscopic characterization of particulate organic matter fractions in soil profiles with imaging VisNIR spectroscopy

**DOI:** 10.1038/s41598-021-95298-8

**Published:** 2021-08-18

**Authors:** Markus Steffens, Lilli Zeh, Derek M. Rogge, Henning Buddenbaum

**Affiliations:** 1 Department of Soil Sciences, Research Institute of Organic Agriculture FiBL, 5070 Frick, Switzerland; 2 Lehrstuhl für Bodenkunde, Department für Ökologie und Ökosystemmanagement, Wissenschaftszentrum Weihenstephan für Ernährung, Landnutzung und Umwelt, Technische Universität München, 85350 Freising-Weihenstephan, Germany; 3 Institut für Bodenkunde und Standortslehre, Technische Universität Dresden, 01737 Tharand, Germany; 4 Hyperspectral-Intelligence Inc, Gibsons, BC Canada; 5 Environmental Remote Sensing and Geoinformatics, Trier University, 54296 Trier, Germany

**Keywords:** Carbon cycle, Environmental monitoring, Agroecology

## Abstract

Organic matter is an important constituent of soils that controls many soil functions and is of vital importance for ecosystem services like climate regulation and food security. Soil organic matter (SOM consists of a wide spectrum of different organic substances that are highly heterogeneous in terms of chemical composition, stability against microbial decomposition and turnover time. SOM is heterogeneously distributed in the soil profile impeding its fast assessment. A technique to accurately measure SOM quality and quantity with a high spatial resolution in the soil profile is presently lacking. Imaging visible light and near infrared spectroscopy (imVisIR) is a promising technique for the fast and spatially resolved assessment of SOM quality and quantity. In this study, we evaluate the potential of imVisIR to quantitatively map the labile particulate organic matter fraction in undisturbed cores from mineral soils.

## Introduction

Organic matter is an important constituent of soil, strongly affecting most chemical, physical and biological soil properties. The pertinence of its accurate quantitative and qualitative assessment will even increase, because of the rising awareness of its role in climate change mitigation and adaptation^1^, and food security. It is widely accepted that soil organic matter (SOM) is not adequately represented purely by its quantity, but that its quality is of equal importance for soil functioning. SOM consists of a wide spectrum of different organic substances varying in bio-chemical and –physical structure and complexity, size, age and turnover time^2^. It contains both undecomposed, large and highly complex plant and animal tissue, and small and microbial-processed molecules in close interaction to the mineral phase. In order to understand and model the impacts of climate change and land use effects, SOM is often defined to be composed of a few conceptual pools with different turnover times^3–5^. The labile pool is the one that is first and mostly affected by human management and climate change^6^. This labile pool is composed of the particulate organic matter^7–9^ (POM)—rather large (> 20 µm), less decomposed remains of plants and animals, that are not stabilized through the intimate interaction with soil minerals but through occlusion in macroaggregates^10^. This stabilization is active for periods of weeks to months, leaving this fraction of SOM relatively prone to decomposition^5,11^. Hence, it is of great importance for nutrient cycling, plant nutrition and sustainable land management. Most authors agree that the stabile SOM pool is represented by the OM that is intimately associated with the mineral phase and thus stabilized for longer periods up to decades and centuries^5,12,13^. This pool is vital for the formation of a stable (micro)aggregate structure and the adsorption capacity of a soil. In the context of climate change and the ongoing discussion if and how carbon sequestration in soils should be considered as a natural carbon sink, a deeper understanding of carbon dynamics and especially the stability and turnover of SOM is of vital importance.

State-of-the-art methods of physical fractionation to derive qualitative and quantitative assessment of SOM is a time- and work-intensive process^14^. Furthermore, this fractionation requires rather large amounts of sample material (> 20 g), which in most studies are taken as a homogenised or sieved sample that represent a diagnostic horizon. This procedure complicates the assessment of SOM dynamics at a high spatial resolution beyond diagnostic horizons, and could even reduce the accurate determination of POM fractions due to the disturbed aggregate structure in the homogenised sample. In addition, the fractionation is a destructive method, preventing additional analyses of the intact soil volume. In order to comprehensively understand, measure, and model the effects of climate and land use change on soils, an accurate and high-resolution assessment at the pedon or soil profile scale is mandatory^15,16^. The importance of high-resolution mapping is even more striking when recalling the recent developments in international agricultural policy, where direct payment for climate change mitigation through agricultural management is discussed (Koronivia Joint Work on Agriculture). Presently, a technique that accurately measures SOM quality and quantity at a high spatial resolution in the soil profile is lacking. Having such a technique that can be automated would enable the evaluation of management and environmental effects on bulk SOM and its more dynamic fractions.

Spectroscopic methods make use of the fact that elements and molecules of simple to complex structure selectively absorb or reflect electromagnetic radiation depending on wavelength. For opaque media like soil, only reflectance spectroscopy can be applied and due to their surface roughness diffuse reflectance is mostly measured. Spectroscopic methods are non-destructive and can be acquired in a fast, robust and easily standardisable process. The interpretation of the data is commonly done by comparing the spectra to standardized spectral libraries or calibrated to other analytical techniques using various statistical approaches. In the visual light (400–700 nm = Vis) and near to shortwave infrared domain (700–2500 nm = NIR), the identification of materials is complex because the signals of different elements and molecules are superimposed. In soil sciences, infrared (IR) reflectance spectroscopy is a standard analytical method to determine various soil properties^17–21^. Many studies have shown that the organic carbon content^22^ and soil texture^23^ can be determined precisely. Fractions^24,25^ and/or chemical composition of SOM^26–28^, mineralogy^29^, and aggregate stability^30,31^ were determined spectroscopically in soil samples. In the context of carbon sequestration and stabilisation, Hermansen et al.^32^ showed even a good predictability of the clay/SOC ratios using VisNIR spectra. In addition to these methodologically interesting studies on small data sets, it could be shown that both the grain size distribution and the organic carbon content can be determined with sufficient accuracy with commercially available VisNIR spectrometers even for the nationwide survey “Bodenzustandserhebung” in Germany^33,34^. Riedel et al. ^35^ prove the potential of VisNIR spectroscopy in a continuous soil monitoring for SOC, pH and various metals on the state level in Germany and Clairotte et al.^36^ describe similar results for France. Several authors showed that the European soil database LUCAS and its new spectral database for Europe^37^ can be used for the comprehensive estimation of soil organic carbon in Europe^38,39^. Viscarra Rossel and Hicks^40^ analysed the potential of VisNIR spectroscopy to estimate total SOC content and stocks and of specific carbon fractions for Australia where they conclude that spectroscopic determination requires minimal sample preparation, is rapid, practical and cheap.

Imaging IR spectroscopy is a rather new spectroscopic application where hyperspectral cameras are used to acquire spatially resolved IR spectra of undisturbed soil cores^41–50^. In previous studies, we could show that imaging VisNIR spectroscopy (further denominated as imVisNIR) can be used to map SOC content^41^ and even discriminate SOM particles with different chemical qualities non-destructively in cores of organic soils with a high spatial resolution^42^. In this study, we evaluate the potential of imVisNIR to quantitatively map the POM fraction in undisturbed cores from mineral soils. We used two hyperspectral cameras to capture the visible (400–1000 nm) and near infrared (1000–2500 nm) reflectance of soil cores from two adjacent sites with similar soil types but different OM inputs due to different grazing intensities. After the soil cores were imaged with the hyperspectral camera, they were sampled and physically fractionated to quantify the amount of POM. The POM fractions were spectroscopically characterized and these spectra were used for image processing and POM identification in hyperspectral images using a supervised classification approach. Using this measurements acquired we have developed an image analysis procedure based on a limited number of spectral features, representing known physical characteristics of the analysed materials. This procedure reduces computing time, and allows for the direct and intuitive interpretation of the results by the user. The successful application of this new technique will allow the rapid and non-destructive assessment of quality, quantity and spatial distribution of the labile OM pool for complete soil profiles. Furthermore, our approach has the potential to significantly improve nationwide soil monitoring systems that rely on core samples^51^ and add to novel soil sampling and analysis systems like the Australian Soil Condition Analysis System (SCANS)^52^.

In order to evaluate the potential of imVisIR and our image analysis procedure, we will test the following hypothesis using two differently grazed plots: (1) The two sampled plots represent similar soil types, are composed of comparable soil constituents, and the SOM is qualitatively similar between the two grazing systems. The ungrazed plot contains more SOM than the grazed plot; (2) the separated SOM fractions show similar spectra between the two plots and a limited number of spectral bands is sufficient to discriminate SOM from mineral soil material and even identify SOM fractions; and (3) imVisIR can be used to quantitatively map the labile OM pool in undisturbed mineral soil profiles.

## Materials and methods

### Study area and soil sampling

Soils were sampled near the Inner Mongolia Grassland Ecosystem Research Station (IMGERS, administered by the Chinese Academy of Sciences; 43°38′ N, 116°42′ E). IMGERS is located in the autonomous region Inner Mongolia (northeastern China) approximately 450 km north of Beijing, near the city of Xilinhot. All soils were classified as Calcic Chernozems^53^, derived from aeolian sediments above acidic volcanic rocks. Generally, secondary carbonates occur in a depth of approximately 30 cm^54^ and downwards, but were not found in the sampled soil volumes.

The study area is located in a gently rolling landscape (average altitude = 1270 m above sea level) and is dominated by *Stipa grandis* and *Leymus chinensis* grasslands. Its climate is classified as a dry and cold middle latitude steppe climate (Bsk). Mean annual temperature and mean annual precipitation are 0.8 °C and 326.5 mm, with the highest values for both between June and August (mean from 1982 to 2003). Before 1979, the whole experimental area was grazed at low intensity. In 1979, plot 1 (24 ha) was fenced and excluded from grazing until now (further denominated as Ungrazed). After 1979, the grazing intensity in the region increased to a moderate level. Plot 2 (24 ha) is grazed by sheep and goats during the whole year, with the highest intensity during summer, equivalent to a grazing intensity of 1.2 sheep units ha^−1^ yr^−1^ (further denominated as Grazed). Further information on the region can be drawn from the synthesis paper of the research group MAGIM^55^.

In 2012, we sampled one soil profile in each plot with a custom-made stainless steel box (100 × 100 × 300 mm^3^). This box was designed to sample an undisturbed, representative volume of a soil profile and allow imaging techniques to be applied on a profile face. The steel box was vertically inserted into the soil from the surface after the litter layer was removed. Both samples contained soil material from the Axh horizons^56^. Then the soil core was carefully excavated and dried in the stainless steel box at 30 °C to a constant weight.

### Imaging setup and pre-processing

Hyperspectral images of the soil profile were taken at the Department of Environmental Remote Sensing and Geoinformatics at the University of Trier. After one image was taken, we sampled the profile face with a grid of 10 squares (2 × 5; each 4.5 × 6 × 1 cm^3^) for further analyses (regions of interest; referred to as “ROI samples”). Then a layer of approximately 15 mm was manually removed from the profile face, the new surface carefully planished and the next image was taken. In total, we took three images and sampled 30 ROIs per core (60 samples in total).

Images of all soil cores were acquired with a HySpex VNIR-1600 (further denominated as VNIR; 410–990 nm) and a HySpex SWIR-320 m-e (further denominated as SWIR; 970–2500 nm) hyperspectral line scanner camera (Norsk Elektro Optikk, Norway). The cameras were set up in a laboratory frame 30 cm above the sample with two tungsten halogen light sources, which are connected via fibre optic cables to two lenses that concentrate the light in a line at the camera’s field of view. This set-up illuminates the sample from a distance of about 35 cm and at an angle of about 45° in front of and behind the camera to reduce shadow and shading. All lamps and cameras were pre-heated for 15 min. The sample was placed on a translation stage, which moved the sample under the cameras, so that images with square pixels were formed from the single lines that the cameras record at a time. The translation speed is automatically aligned to the optimum integration time by the HySpex software. Prior to each experiment the integration time was adjusted by increasing it until we got over-saturation on a grey reference and then decreasing it by 5%. In the direction perpendicular to the movement of the sample (across-track direction), the VNIR camera recorded 1600 pixels with a field of view of 17° and the SWIR camera 320 pixels with a field of view of 14°. The instantaneous field of view for each pixel was 0.18 mrad across the track and 0.36 mrad along the track for VNIR and 0.73 mrad for SWIR. The area that was recorded by the camera from 30 cm distance was 10 cm wide, so that a single pixel was 62.5 µm wide in VNIR and 250 µm in SWIR. A soil profile of 30 cm in length consisted of 4800 image lines in VNIR and 1200 lines in SWIR. The spectral bands were recorded in the spectral range of 410 to 990 nm with a spectral sampling distance of 3.7 nm and from 970 to 2500 nm with a sampling distance of 5.5 nm. The VNIR and SWIR data were recorded in 12-bit and 14-bit radiometric resolution, respectively^57^, and the dark current was automatically removed by the HySpex software. A certified reflectance standard white reference panel of known reflectivity (Spectralon) was recorded with each image. The object reflectance was calculated for each image line (along track) separately following Eq. (1) because the illumination was not perfectly uniform across the whole sample:

1 $${\varvec{\rho}}_{{{\varvec{obj}}}} = \frac{{{\varvec{L}}_{{{\varvec{obj}}}} }}{{{\varvec{L}}_{{{\varvec{ref}}}} }} \times {\varvec{\rho}}_{{{\varvec{ref}}}}$$

where *L*_obj_ is the radiance from the object in camera units, *L*_ref_ is the radiance from the white reference panel and *ρ*_ref_ is the reflectance of the white reference panel^49,58,59^. All images were spatially and spectrally resampled to increase the signal-to-noise ratio. By calculating the mean value of *n* pixels, the signal to noise ratio can be improved by √*n*. Furthermore, by taking the mean value of several neighbouring spectral bands, the signal to noise ratio is also improved. Thus, we resampled all three images spatially and spectrally to increase the signal to noise ratio: four pixels were combined to one and the spectral bands were resampled from 3.7 to 10 nm bands. The third layer in both cores showed artefacts in the SWIR > 2300 nm that obstructed the data evaluation. The artefacts were not associated to natural phenomena like different water contents or variations in illumination and could not be considered in the following data analyses. Therefore, we discarded the third layer in both cores and did all image analyses just on the first and second layer. All image processing and analyses were conducted in the software ENVI version 4.8 (Exelis Visual Information Solutions, Boulder, Colorado, USA). All statistical tests (ANOVA with the least significant difference test as post hoc test) were carried out using SPSS 19 (IBM, Armonk, USA).

### Qualitative and quantitative characterization of soil samples

Referring to the procedure described in Steffens et al.^60^, all 60 air-dried samples were dry-sieved to 2 mm and afterwards separated by a physical fractionation procedure in order to obtain soil fractions that represent distinct SOM pools^14,61,62^. The free particulate organic matter (fPOM) was separated with a Sodium polytungstate solution (*ρ* = 1.8 g cm^-3^). The floating fPOM was extracted by aspiration with a water jet pump. For the determination of the occluded particulate organic matter fraction (further denominated as oPOM), the heavy fraction (> 1.8 g cm^-3^) was treated by ultrasonication. An energy input of 150 J ml^-1^ was applied to disrupt all macro-aggregates and to obtain the greatest similarity of clay yields compared with standard particle size analysis, but also to minimize the production of artefacts following intensive ultrasonication^63^. With a subsequent density fractionation step (Sodium polytungstate solution, *ρ* = 1.8 g cm^-3^), the oPOM floating on the suspension was obtained after centrifugation (10 min at 4000 g). To remove the Sodium polytungstate from all POM fractions, the samples were washed with de-ionized water over a 20 µm sieve and the remaining filtrate was then passed through a 0.22 µm filter to collect a small oPOM fraction (further denominated as soPOM), which was subsequently washed with de-ionized water. The remaining sediment (further denominated as MinRest) was centrifuged (20 min at 4000 g) and washed with de-ionized water several times in order to remove excessive salt.

All bulk soils and physical soil fractions were dried at 40 °C after washing, ground and analysed in duplicate for organic carbon (OC) and total nitrogen concentrations by dry combustion on a EuroEA elemental analyser (Hekatech GmbH, Wegberg, Germany). All samples were free of carbonates, so that the total C concentration equalled the OC concentration.

For each ground sample (60 bulk soil and 240 physical soil fraction), we collected reflectance spectral measurements (over the 350–2500 nm wavelength range with 2151 bands at a spectral resolution of 1 nm with an average band sampling interval of ~ 2 nm) using the ASD (Analytical Spectral Devices Inc., Boulder, CO, USA) FieldSpec-Pro spectroradiometer that was equipped with an 8°-fore optic. The sample measurements were acquired at the Land Surface Department (German Aerospace Center) spectroscopy lab with constant illumination conditions from two Quartz halogen lamps (300 W each) at a zenith angle of 30°. All samples were measured in triplicate and the spectra were smoothed using a three-band sliding mean to reduce noise. Wavelength resampling was applied to match the HySpex wavelengths to the VNIR and SWIR cameras using the standard ENVI resampling tool.

## Results and discussion

### Partitioning of soil organic matter in physical fractions in steppe soils as affected by grazing

**Figure 1:**
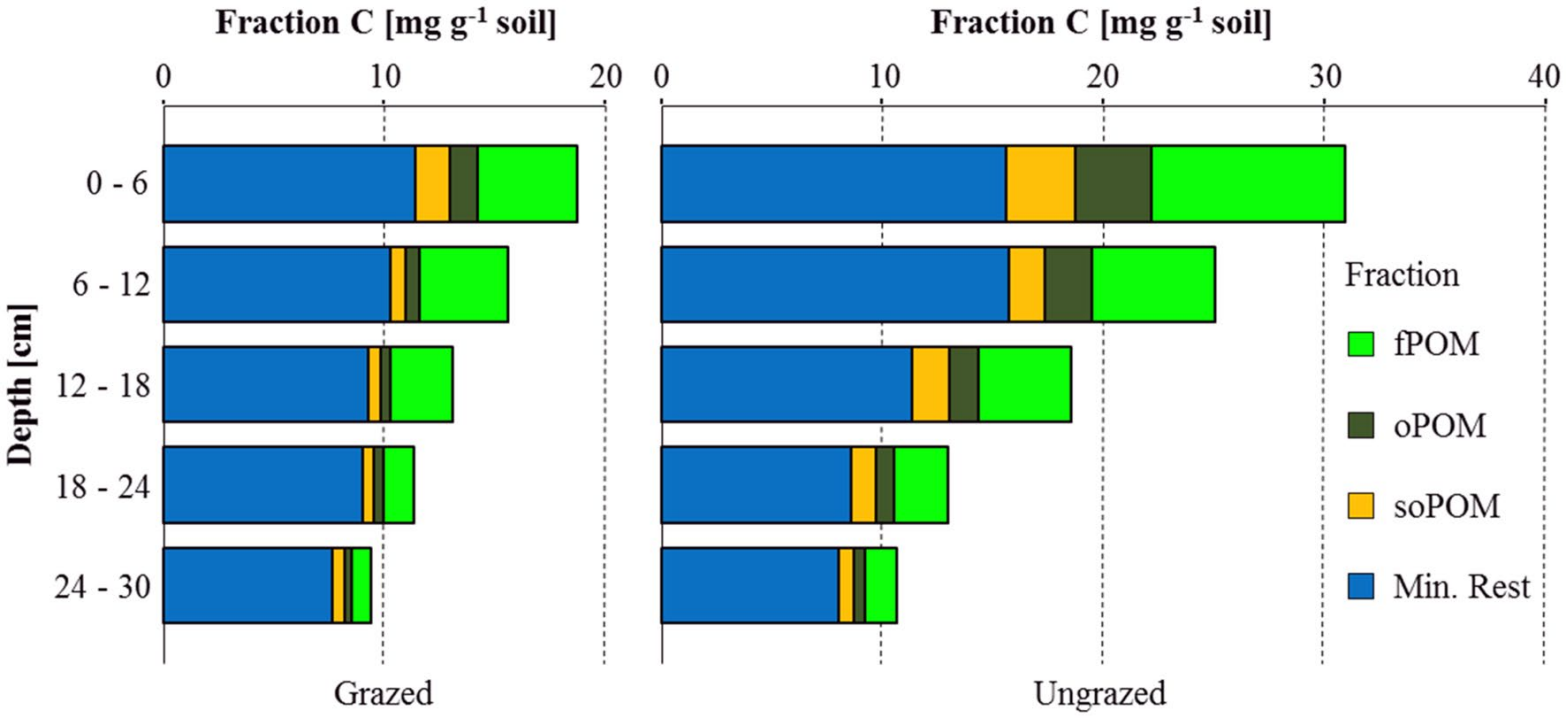
Partitioning of organic carbon between different physical fractions in grazed and ungrazed soil profiles. The contributions of the four physical fractions in each plot and depth increment sum up to the total organic carbon content of the respective layer. Differences to the bulk soil organic carbon content are losses due to physical fractionation (compare Table 1). Each box gives the mean of four fractionated samples (two profiles per plot × two blocks per sampling depth).

**Figure 2:**
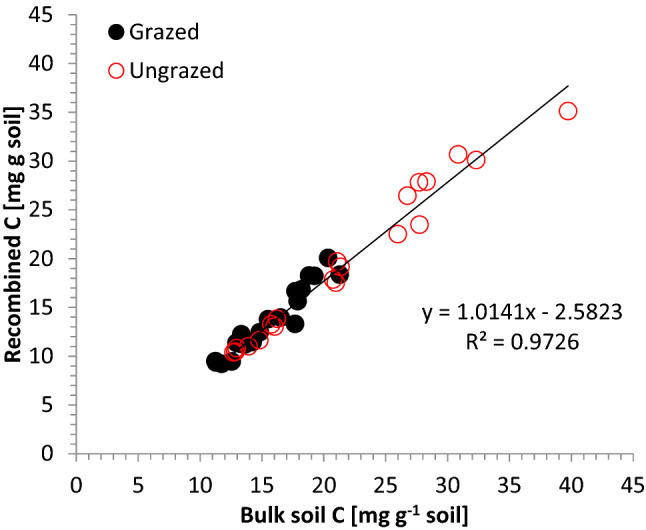
Scatterplot between bulk soil organic carbon content and the recombined fractions, displaying the recovery of the physical fractionation procedure.

The ungrazed soil core contained significantly more SOM than the grazed core in the two uppermost sampling depths (0–6 cm and 6–12 cm; Table 1 and Figs. 1 and 2). This applied for the total content of SOM and for each individual fraction. In the third sampling depth (12–18 cm), fPOM and oPOM were still significantly higher in the ungrazed core, but we found no significant differences for the soPOM and the MinRest fractions. In the fourth sampling depth (18–24 cm), only fPOM was higher in the ungrazed core, while in the fifth depth (24–30 cm), no significant differences could be found at all. We explain the difference between the plots with two processes—first, long-term grazing cessation leads to significantly higher amounts of SOM in the ungrazed plot because of the higher above- and belowground biomass production due to grazing cessation; and second, more deposition of wind-blown material around the recovering vegetation in the ungrazed plot^60,64^. Differences between the two plots are pronounced in the uppermost centimeters of the soil profile and diminish with increasing sampling depth where we assume that the higher biomass production of the recovering vegetation following grazing cessation leads to higher litter inputs to the topsoil. This process takes longer and needs changes in the species composition until wider and deeper ramified rooting system are established and deeper soil layers are affected by higher biomass inputs in the ungrazed plot.

**Table 1:** Characteristics of physical soil organic matter fractions (PhysPOM) and spectroscopically identified organic matter (SpecPOM) fractions.

Plot		Grazed					Ungrazed				
Depth	(cm)	0–6	6–12	12–18	18–24	24–30	0–6	6–12	12–18	18–24	24–30
Bulk soil	(mg C g^-1^ fraction)	19.9 ± 1.1	17.9 ± 0.2	15.0 ± 1.3	13.5 ± 0.6	11.7 ± 0.6	32.8 ± 4.9	27.0 ± 0.8	21.0 ± 0.2	15.7 ± 0.6	13.1 ± 0.6
fPOM		68.6 ± 14.7	26.8 ± 6.6	24.0 ± 1.0	22.4 ± 2.6	22.7 ± 3.2	102.4 ± 12.4	56.0 ± 25.7	29.5 ± 4.9	21.6 ± 4.1	25.3 ± 7.2
oPOM		363.5 ± 27.9	286.3 ± 43.6	288.9 ± 18.6	285.2 ± 39.5	308.0 ± 12.1	431.8 ± 25.3	416.6 ± 12.4	380.9 ± 22.6	378.5 ± 8.0	385.7 ± 41.9
soPOM		229.7 ± 50.1	164.8 ± 118.2	222.0 ± 17.8	221.4 ± 115.4	200.6 ± 101.4	231.6 ± 36.8	217.6 ± 36.6	234.7 ± 52.4	247.5 ± 19.9	244.5 ± 105.5
Min. Rest		12.9 ± 0.7	12.8 ± 0.7	11.2 ± 0.2	10.3 ± 0.7	8.6 ± 0.3	18.1 ± 2.2	18.7 ± 1.8	14.3 ± 0.5	10.5 ± 0.6	9.3 ± 0.6
Bulk soil	C/N	10.4 ± 0.3	10.3 ± 0.1	10.2 ± 0.2	10.1 ± 0.1	10.0 ± 0.1	10.9 ± 0.5	10.5 ± 0.1	10.1 ± 0.1	10.1 ± 0.1	10.1 ± 0.2
fPOM		14.9 ± 1.1	11.2 ± 1.0	11.4 ± 0.2	11.1 ± 0.6	13.0 ± 1.1	16.0 ± 1.6	13.6 ± 1.3	11.3 ± 0.6	10.7 ± 0.9	12.1 ± 0.6
oPOM		16.8 ± 0.3	18.4 ± 0.9	20.1 ± 1.8	18.9 ± 1.2	18.9 ± 1.3	14.5 ± 0.2	16.9 ± 0.2	17.1 ± 0.5	18.3 ± 1.1	18.2 ± 0.6
soPOM		14.1 ± 1.0	12.2 ± 2.4	13.5 ± 1.5	13.6 ± 1.3	14.5 ± 0.7	12.6 ± 0.2	12.3 ± 0.4	12.2 ± 0.5	13.2 ± 0.2	12.9 ± 0.6
Min. Rest		8.3 ± 0.1	8.5 ± 0.3	8.4 ± 0.2	8.2 ± 0.3	8.1 ± 0.2	8.8 ± 0.2	9.0 ± 0.1	8.5 ± 0.1	8.2 ± 0.1	8.0 ± 0.2
fPOM	(mg C g^−1^ soil)	4.5 ± 0.6^a^	4.0 ± 1.9^a^	2.8 ± 0.3^ab^	1.3 ± 0.1^bc^	0.9 ± 0.1^c^	8.8 ± 2.6^a*^	5.6 ± 0.3^b^	4.2 ± 0.8*^bc^	2.4 ± 0.3*^cd^	1.4 ± 0.4^d^
oPOM		1.3 ± 0.3^a^	0.6 ± 0.4^b^	0.4 ± 0.3^b^	0.5 ± 0.4^b^	0.3 ± 0.1^b^	3.4 ± 0.6^a*^	2.1 ± 0.4*^b^	1.3 ± 0.3*^c^	0.8 ± 0.1^cd^	0.5 ± 0.2^d^
soPOM		1.6 ± 0.4^a^	0.6 ± 0.6^b^	0.6 ± 0.5^b^	0.5 ± 0.4^b^	0.6 ± 0.3^b^	3.2 ± 0.9^a*^	1.6 ± 0.4*^b^	1.7 ± 0.9^bc^	1.1 ± 0.5^b^	0.7 ± 0.5^b^
Min. Rest		11.4 ± 0.6^a^	10.3 ± 1.0^ab^	9.3 ± 0.2^bc^	9.0 ± 0.5^c^	7.7 ± 0.3^d^	15.6 ± 2.0^a*^	15.7 ± 2.2*^a^	11.4 ± 0.4*^bc^	8.6 ± 0.5^c^	8.1 ± 0.6^c^
fPOM + oPOM		5.8 ± 0.6^a^	4.7 ± 2.2^ab^	3.3 ± 0.6^bc^	1.8 ± 0.4^c^	1.2 ± 0.1^c^	12.2 ± 2.3^a*^	7.7 ± 0.2*^b^	5.5 ± 0.7*^c^	3.3 ± 0.3^d*^	1.9 ± 0.5^d*^
POM total		7.3 ± 0.8^a^	5.3 ± 2.6^ab^	3.8 ± 1.0^bc^	2.3 ± 0.5^c^	1.7 ± 0.3^c^	15.4 ± 2.4^a*^	9.3 ± 0.5*^b^	7.2 ± 0.8*^bc^	4.4 ± 0.5^c*^	2.6 ± 0.8^e^
PhysPOM Recovery	(Mass%)	96.3 ± 1.0	95.2 ± 1.8	95.6 ± 0.6	94.7 ± 0.5	93.6 ± 0.1	96.9 ± 0.6	96.8 ± 0.4	95.1 ± 0.4	94.2 ± 0.4	93.5 ± 0.3
	(C%)	94.3 ± 5.5	87.5 ± 8.5	87.5 ± 3.7	83.9 ± 3.3	80.4 ± 4.2	95.0 ± 5.2	92.8 ± 8.2	88.3 ± 4.3	82.5 ± 3.0	81.6 ± 1.8
SpecPOM	(%)	3.3 ± 2.6^a^	1.2 ± 0.3^ab^	0.7 ± 0.2^b^	0.2 ± 0.0^b^	0.2 ± 0.1^b^	17.0 ± 3.3^a*^	2.3 ± 1.0^b^	0.8 ± 0.3^b^	0.4 ± 0.3^b^	0.1 ± 0.1^b^
	(Pixels)	22.4 ± 9.3^a^	12.0 ± 1.4^b^	11.6 ± 2.4^b^	7.9 ± 1.4^b^	6.9 ± 1.4^b^	240.5 ± 254.4^a^	17.6 ± 4.4^a^	12.0 ± 5.5^a^	12.8 ± 5.1^a^	7.5 ± 1.2^a^

Characteristics are given as mean values and standard deviations and were calculated from four data points (two profiles per plot × two blocks per sampling depth in each profile). The first section shows the carbon concentrations, the second section the C/N ratio, and the third section the carbon stocks as calculated from carbon concentrations and masses. Different letters in the third and fifth section show significant differences between the five sampled depths for the respective plot (ANOVA with the least significant difference test as post hoc test). Asterisks in the third and fifth sections show significantly different numbers between the two plots in the respective fraction and sampling depth.

In addition, these findings fit well to the idea that the different SOM fractions represent pools with different turnover times^14,16,65,66^. Fresh OM inputs enter the system in the form of litter, which is first physically broken down to smaller particle sizes (fPOM). At a given step during their continuous decomposition, these particles will be small enough to be included in macroaggregates, during the continuous formation and break-up of soil aggregates. This fraction of SOM particles can be isolated via physical fractionation as macro oPOM. With time and continuous aggregate turnover cycles, these particles are further broken down in size and chemically altered through lysis of easily decomposable molecules by microbial enzymes. These particles are continuously occluded in smaller aggregates and at one point become smaller than 20 µm and not being part of the POM anymore due to the sieve cutoff of 20 µm (soPOM). It takes longer than 33 years after grazing cessation to increase the amount of oPOM and soPOM in soil layers deeper than 12 and 18 cm, and even longer below 24 cm. These findings corroborate other studies that were conducted in the same long-term field experiment at IMGERS^16,60,67^, and the widely accepted impacts of grazing on SOM in semiarid grasslands^68^.

We assume these soil cores to be representative for the two plots with different grazing intensities and to be ideal test objects for the hyperspectral assessment of various SOM fractions in undisturbed soil cores.

### Spectroscopic characterization of particulate organic matter fractions

**Figure 3:**
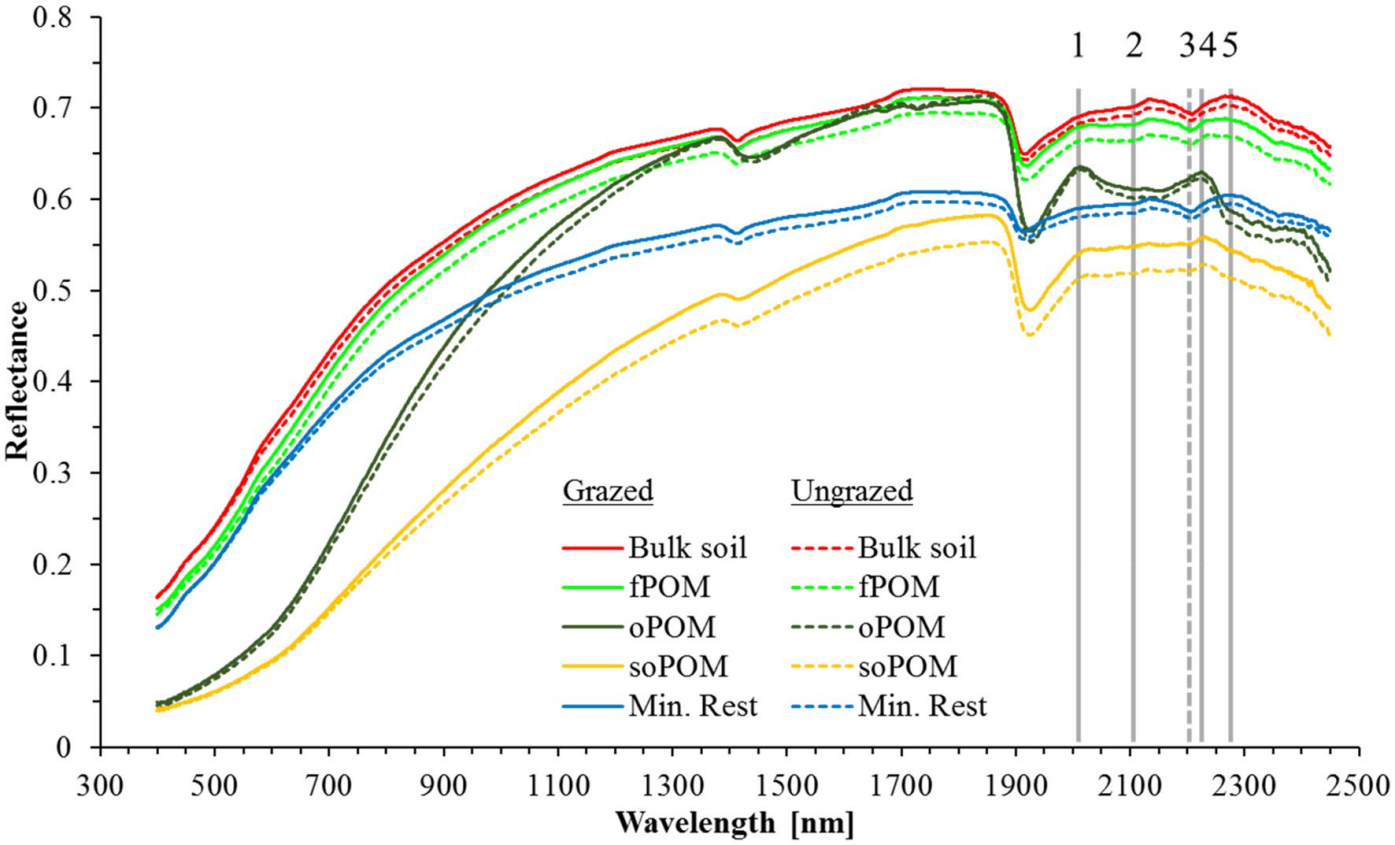
Mean VisNIR reflectance spectra of bulk soils and four physical organic matter fractions from grazed and ungrazed soil profiles. Each spectrum gives the mean of 20 samples (two profiles per plot × five sampling depths per profile × two blocks per sampling depth). The five vertical lines illustrate the five bands used for the identification of the SpecPOM using a constrained unmixing approach (Solid lines 1, 2, 4, and 5 identify absorption features characteristic for organic substances and the dashed line 3 marks a typical feature of phyllosilicates).

The spectra of the different physical fractions are similar across sampling depths and both plots (Fig. 3). This corroborates hypothesis #2 that the two plots represent the same soil type, are composed of similar soil constituents, and the OM inputs are qualitatively similar between the two grazing systems. The applied fractionation method successfully extracted qualitatively similar fractions from both profiles. Clearly different spectra were measured for the different physical fractions. The bulk soil samples showed typical, but rather featureless spectra. Their Vis part is characterised by a typical soil reflectance spectrum with a steady convex shape in the visible part and a plateau with water absorption features at 1450 and between 1920 and 1940 nm^18^. The only distinct feature occurs at 2206 nm (line 3) and marks the characteristic adsorption of the Al-O-bond in the octahedra of phyllosilicates^69^. We were surprised to find exactly the same spectra for the fPOM fraction, because this fraction should be composed only of organic matter. We explain this contradiction with the large amount of mineral soil material sticking to the fPOM particles. The fPOM fraction is separated from the bulk sample during the first fractionation step. In this step, the bulk sample is put in the density solution and the floating material is removed with the waterjet pump the other day. Intimate associations between large OM particles and adhering soil particles are not yet broken by the subsequent sonication step as it happens to oPOM particles. Proof can be found in the low OC concentrations of the fPOM fraction (Table 1). The OC concentration of the fPOM varies between 21.6 and 102.4 mg*g^−1^ and therefore much below the expected 450 mg g^−1^ of pure plant material^2^. This explains the featureless and soil-like spectra of the fPOM fraction. Furthermore, this finding underlines the high biological activity in Chernozems, the typical soils of semi-arid grasslands. OM is incorporated mostly through bioturbation resulting in a dark-coloured thick Axh-horizon. During this incorporation step, the fresh OM is intimately mixed with the mineral soil material. Further proof for this explanation of the soil-like spectra of the fPOM fraction can be found in the similar spectra of the MinRest fraction. The only obvious difference between bulk soil, fPOM, and MinRest is the overall lower reflectance of the latter. We assume the smaller particle size of the MinRest, in consequence of sonication that disrupted most macro- and many micro aggregates, results in an overall smaller particle size of the MinRest and therefore higher absorbance due to total internal reflection.

We found clearly different spectra and more pronounced spectral features for the oPOM and soPOM fractions. Both fractions showed a concave spectrum with a clearly lower reflectance between 350 and 1000 nm, no absorbance at the characteristic phyllosilicate feature at 2206 nm, but a typical feature of cellulose and lignin between 2000 and 2300 nm^70^. This feature is pronounced for the oPOM, but only weakly discernible for the soPOM fraction. We explain this difference through the higher degree of decomposition of the soPOM^16^, the resulting higher absorption at smaller particles^42^, and finally lower signal-to-noise ratios. These features are characteristic for organic matter and explain the iconic dark colour of the humus. The purity of the oPOM fraction is underlined by its high OC concentrations of up to 431.8 mg*g^−1^, being in the range of pure plant material. These numbers and spectra underline the earlier findings of Ben-Dor et al.^70^ during a controlled decomposition experiment and its effects on the VisNIR reflectance of two types of organic matter.

Our results show that the applied fractionation routine separates meaningful and reproducible SOM fractions from various soil samples taken from different plots and various sampling depths. In addition, VisNIR spectroscopy shows clearly different spectra for these fractions and can be used to distinguish these different SOM fractions. We ask for more studies to test this finding in other soil systems and check if the different fractions show similar spectral features.

### Mapping of particulate organic matter in steppe soils with imaging VisNIR spectroscopy

The previous chapters show that the selected soil cores contain different amounts of SOM because of different grazing intensities and that different SOM fractions can be discriminated by characteristic spectral features in the VisNIR. We still assume three main challenges to complicate the POM mapping attempt: (1) the abundance of POM is much lower in mineral horizons compared to organic surface layers; (2) POM particles are smaller in mineral soil horizons and thus it is more likely that one pixel not only contains organic or mineral materials; and (3) POM particles can be partly or completely covered by mineral materials. All three points contribute to the fact that most pixels will show mixed spectra with different contributions of organic and mineral materials, hampering a simple pixel-wise classification. In order to quantify the amount of POM in mineral soil horizons, we applied a constrained linear unmixing to quantify the contribution of organic and mineral materials to each pixel’s spectrum. We used the mean ASD spectra of the MinRest and the oPOM fractions as the endmember for this unmixing because these fractions were the purest mineral and organic fraction in this study. The spectroscopically identified organic fraction is further denominated as SpecPOM in contrast to the physically fractionated PhysPOM.

The high spectral resolution of the applied hyperspectral cameras allow for precise qualitative and quantitative image interpretation. State-of-the-art regression algorithms try to benefit from this information and yield as much explanatory power as possible. We found that especially for imaging spectroscopy with its millions of spectra available in one image, overfitting can easily occur pretending a higher accuracy^41^. To overcome this problem, we selected only five out of the available 416 spectral bands for the unmixing approach. We selected the wavelengths 2012 nm, 2108 nm, 2228 nm, and 2276 nm as identifiers of organic matter, and the wavelength 2204 nm as an identifier for mineral materials based on the results of the ASD measurements (Fig. 3) and a literature review^17,18,71–73^.

**Figure 4:**
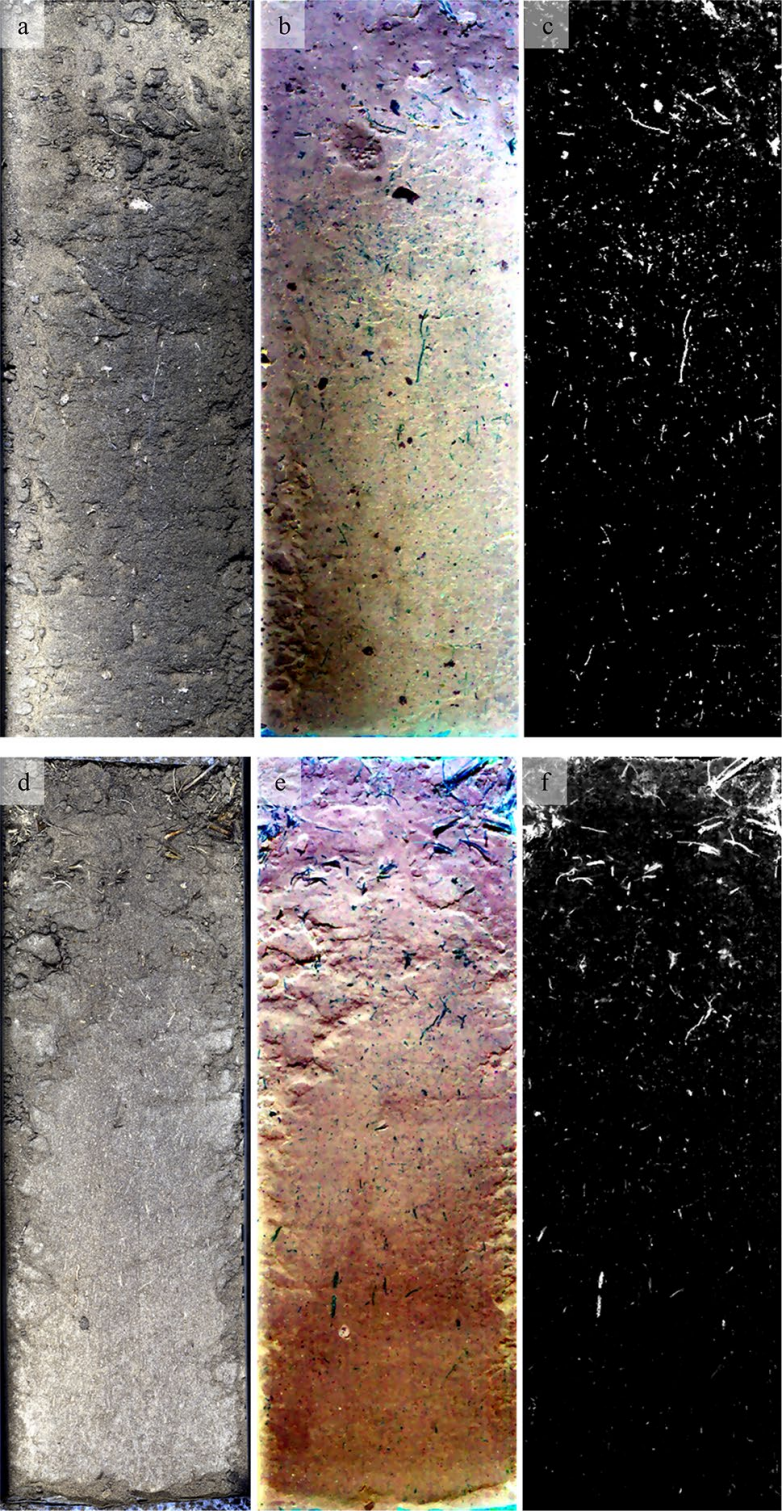
True color images of the VNIR-1600 (**a** and **d**; red: 660 nm, green: 560 nm, blue: 480 nm), false color images of the SWIR-385 (**b** and **e**; red: 2276 nm, green: 2204 nm, blue: 2012 nm; corresponding to the lines 5, 3, and 1 in Fig. 2), and fractional cover of the organic fraction for the soil profiles grazed 2 and ungrazed 2 (**c** and **f**; bright pixels show a high contribution of organic matter). Both profiles are 30 cm long and 9 cm wide.

Figure 4(c and f) shows the resulting fractional abundance maps for two out of four soil profiles. The brighter a pixel is, the higher is the relative contribution of organic matter to the respective spectrum or more area of the pixel is covered by organic matter. All four profiles show higher contributions of organic matter in the topsoil and decreasing contributions in deeper sections of the soil profiles. In Table 1, the line SpecPOM reports the mean relative contribution of organic matter for all ROIs. It shows that most pixels in all profiles were dominated by mineral material, but the two uppermost ROIs of the ungrazed profiles show a significantly higher (< 0.01) contribution of the organic fraction compared to the lower ROIs. The next deeper two ROIs still show higher contributions of the organic fraction in the ungrazed profiles, but at a lower level of significance of 0.03. The grazed plot shows a similar vertical distribution but less distinct differences between the sampled ROIs. This vertical distribution fits nicely to the findings from physical fractionation (Table 2). We found the highest coefficients of correlation between the relative contribution of SpecPOM and the amount of the oPOM fraction as well as the sum of all POM fractions combined when all four profiles were considered. The correlations were lower when only the two grazed profiles were analysed. We assume that beside the generally lower SOM content in the grazed plot, smaller POM particles due to animal trampling reduce precision of SpecPOM identification. These findings already verify our third hypothesis and support the applicability of imVisIR for the quantification of POM fractions in soil profiles.

**Table 2:** Correlation matrix between the spectroscopically identified particulate organic matter (SpecPOM) and the contribution of physically extracted fractions of soil organic matter for each of the 40 blocks (2 plots × 2 profiles per plot × 2 blocks per depth × 5 sampling depths).

	Plot	Bulk soil	fPOM	oPOM	soPOM	Mineral rest	POM	fPOM + oPOM
SpecPOM	All	0.76	0.73	0.82	0.72	0.65	0.80	0.79
	Grazed	0.72	0.50	0.46	0.36	0.66	0.52	0.52
	Ungrazed	0.75	0.76	0.86	0.77	0.61	0.84	0.82
SpecPOM size	All	0.43|0.69	0.39|0.74	0.54|0.55	0.41|0.50	0.46|0.63	0.46|0.70	0.31|0.57
	Grazed	0.69	0.62	0.55	0.64	0.59	0.68	0.36
	Ungrazed	0.38|0.78	0.37|0.83	0.53|0.60	0.38|0.43	0.44|0.70	0.44|0.75	0.38|0.75

POM is the sum of the three fractions fPOM, oPOM and soPOM. Pearson’s coefficients of correlation are given for all 40 blocks combined, (“All”) and for the grazed and the ungrazed soil profiles individually. We show the coefficients for the area covered by SpecPOM and the mean size of the SpecPOM particles. The correlations for SpecPOM size and both treatments combined (“All”) are given for all 40 data points (first value) and with two outliers in the first layer of the ungrazed profile excluded (second value; size of 376.96 and 530.85 pixels).

**Figure 5:**
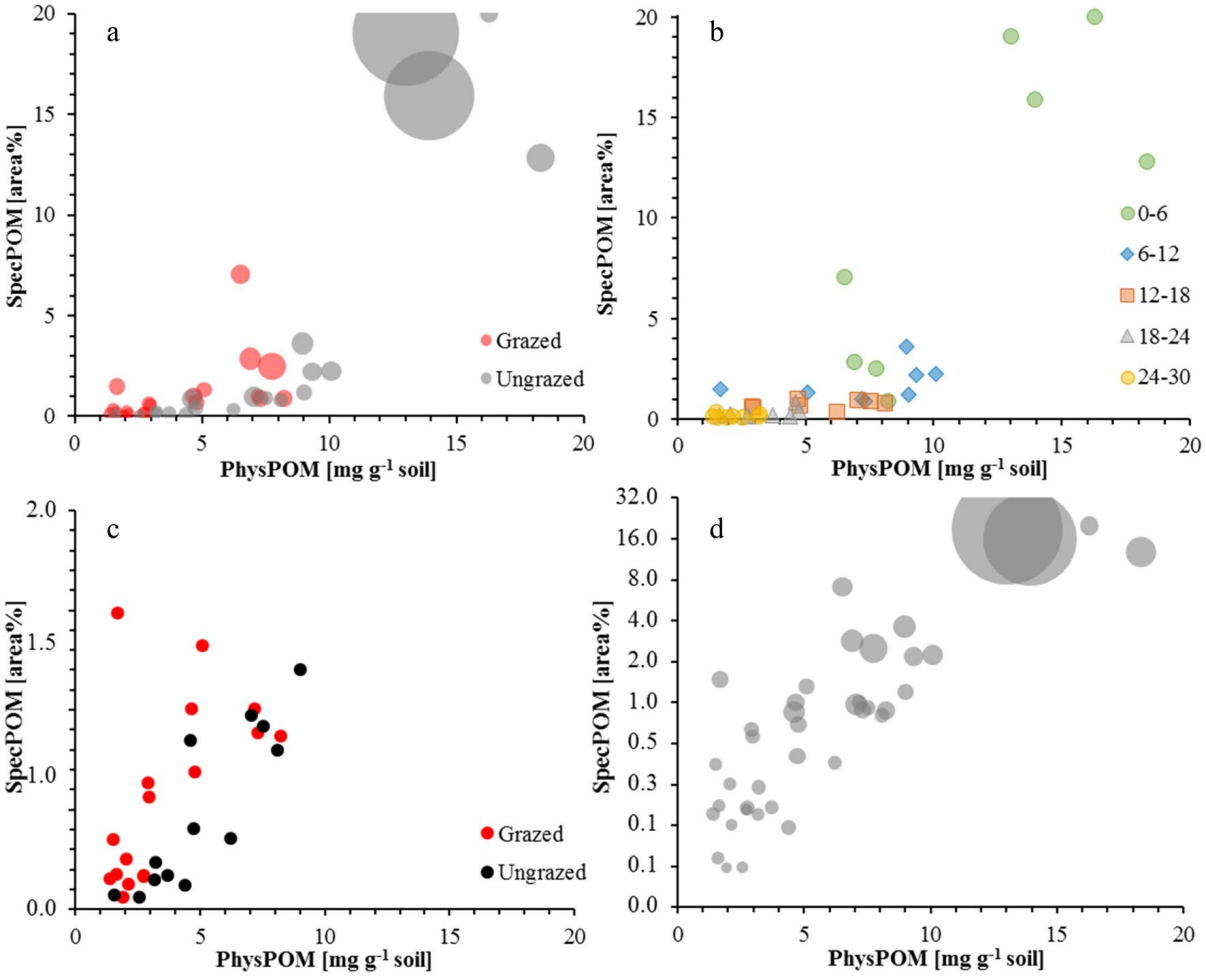
Scatter plots illustrating correlations between the amount of organic carbon in physically isolated particulate organic matter fractions (PhysPOM) and spectroscopically identified organic matter dominated pixels in hyperspectral images (SpecPOM). Bubblesize in parts a and d correspond to the size of the organic particles as identified in the hyperspectral images using segmentation images. Part a shows the correlation as affected by management, part b as affected by sampling depth, part c shows the correlation as affected by management without the four 0–6 cm samples from the ungrazed profiles, and part d illustrates the correlation for all samples as affected by management with a log-scaled y-axis for the SpecPOM.

More information to comprehensively assess and map the current state of SOM in an undisturbed soil core can be seen using imVisIR. Continuing from this point, the fractional abundance maps in Fig. 4 show that the POM is not homogeneously distributed across the ROIs but accumulated in patterns. It is noteworthy that the organic matter-dominated pixels are mostly clustered in predominately longish structures reflecting typical shapes of OM particles like roots and undecomposed leaves. In order to measure the size of these SpecPOM particles, we analysed the connectivity of pixels with high fractional abundances of organic matter by calculating segmentation images. This approach produces information on the number of directly connected pixels with fractional abundances of organic matter > 5%. In Table 1, SpecPOM [pixels] gives the mean SpecPOM size for each ROI. Especially in the two uppermost ROIs the ungrazed plot reveals significantly higher particle sizes. This is in line with the accrual of POM as a consequence of the long-term ungrazing and a significant accumulation of plant litter and supports our assumption of larger particle sizes in the ungrazed plot. Figure 5 gives a scatterplot of the content of PhysPOM (sum of fPOM, oPOM and soPOM) and the relative abundance of SpecPOM for all ROIs. SpecPOM particle size is included in Fig. 5a as bubble size. By combining Fig. 5a and b, it is clear that the four highest values represent the uppermost two ROIs in the two ungrazed profiles. From this figure we deduce that there is no global linear correlation between PhysPOM and SpecPOM and that the particle size is of great importance for the quality of the SpecPOM assessment. Underestimation due to small POM particles in lower layers is as likely as overestimation in topsoils. In future studies, special attention should be placed on the assessment of POM size and the spatial resolution of hyperspectral cameras. In addition, we ask for studies evaluating the robustness and universal applicability of the five selected spectral bands for the identification of POM particles across different soil types.

**Figure 6:**
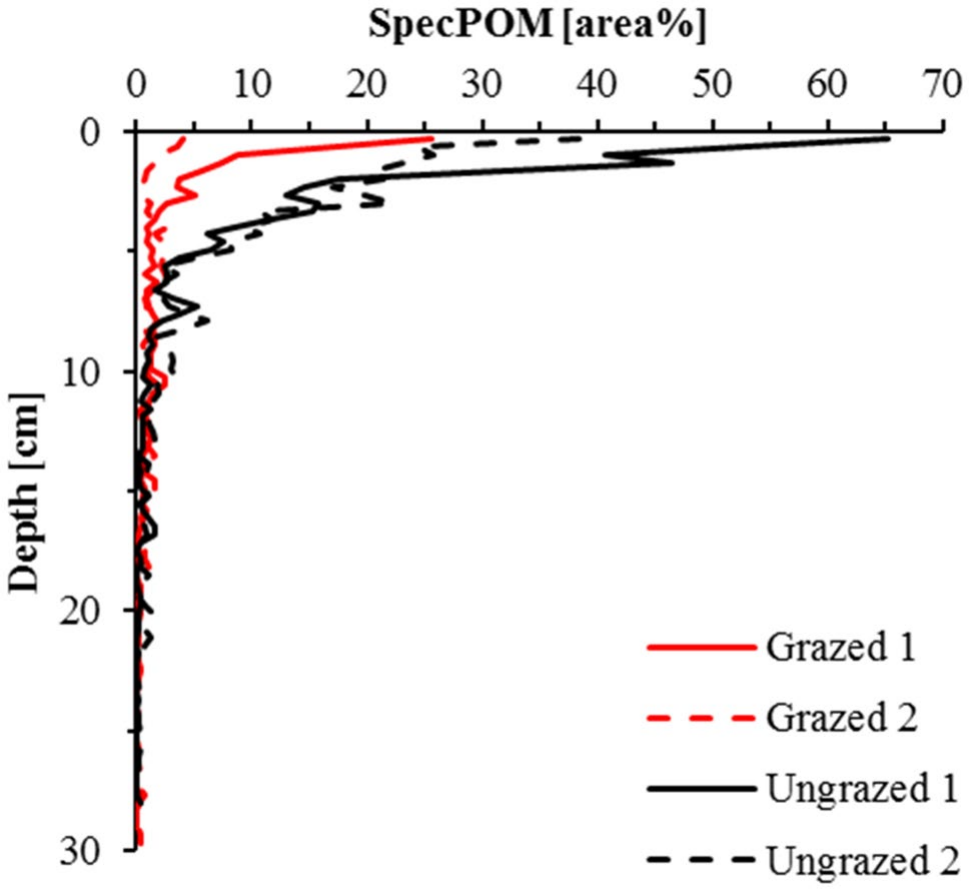
Linewise fractional cover of the SpecPOM for two grazed and two ungrazed soil profiles (summed fractional cover for 10 image lines).

imVisIR provides a much higher spatial resolution than the minimum sampling area for physical fractionation. We calculated the fractional cover for each image line to illustrate the power of imVisIR (Fig. 6) to elucidate SOM dynamics along a soil profile. This figure shows that already the first six cm inherit a high variability of the area covered by SpecPOM. Classic soil analyses (have to) consider horizons to be relatively homogeneous. We assume that imVisIR can determine changing qualities and quantities of SOM in soils due to management or climate faster than time- and work-intensive physical fractionation. This approach could also be used as a preprocessing step to mask areas of high POM contents and enable the better estimation of mineral-associated organic matter. In order to proof this future application, studies should test if POM fractions in different soil types and under various management systems are spectrally comparable, too.

We conclude that imVisIR has a proven potential to quantitatively map SOM and its specific fractions in undisturbed soil cores with a high spatial resolution. This perception opens new avenues in (1) earlier assessing management effects on SOM compared to state-of-the-art fractionation techniques, and (2) elucidating SOM dynamics considering spatially resolved processes like plant-soil- and rhizosphere interactions, bioturbation and tillage. We see large potential for the application of this technique in precision agriculture with adapted fertilization systems, intensive and nation-wide soil monitoring systems that rely on soil core sampling, and comprehensive soil carbon accounting systems that operate in the context of carbon credits and climate change mitigation.
